# Characterization of Primary IGF-1 Deficiency in a Cohort of Canadian Children with Short Stature Using a Novel Algorithm Tailored to Electronic Medical Records

**DOI:** 10.3390/children11060727

**Published:** 2024-06-14

**Authors:** Rinila Haridas, Carly Baxter, Saunya Dover, Ellen B. Goldbloom, Ivan Terekhov, Marie-Eve Robinson

**Affiliations:** 1 Children’s Hospital of Eastern Ontario Research Institute, Ottawa, ON K1H 8L1, Canada; rinila.haridas@ubc.ca (R.H.); carly.baxter@dal.ca (C.B.); sdover@cheo.on.ca (S.D.); egoldbloom@cheo.on.ca (E.B.G.); iterekhov@cheo.on.ca (I.T.); 2 Division of Endocrinology & Metabolism, Children’s Hospital of Eastern Ontario, Ottawa, ON K1H 8L1, Canada; 3 Department of Pediatrics, Faculty of Medicine, University of Ottawa, Ottawa, ON K1H 8L1, Canada

**Keywords:** severe primary IGF-1 deficiency, electronic medical record, short stature, pediatric, electronic algorithm

## Abstract

(1) Background: Severe primary insulin-like growth factor-I deficiency (SPIGFD) is a rare disorder causing short stature in children due to low insulin-like growth factor 1 (IGF-1) levels. Given the sparsity of reported cases of SPIGFD worldwide, the condition may be underdiagnosed, potentially preventing affected children from receiving therapy with recombinant human IGF-1 (rhIGF-1). Our objective was to determine the prevalence of SPIGFD among children with short stature at a large pediatric tertiary care center through the use of a novel electronic medical record (EMR) algorithm. (2) Methods: We queried our EMR using an algorithm that detected all children seen at our center between 1 November 2013 and 31 August 2021 with short stature and low IGF-1. We then conducted chart reviews, applying established diagnostic criteria for those identified with potential SPIGFD. (3) Results: From a cohort of 4863 children with short stature, our algorithm identified 30 (0.6%) patients with potential SPIGFD. Using chart reviews, we determined that none of these patients had SPIGFD. (4) Conclusions: Our algorithm can be used in other EMRs to identify which patients are likely to have SPIGFD and thus benefit from treatment with rhIGF-1. This model can be replicated for other rare diseases.

## 1. Introduction

Growth hormone (GH) is essential for growth in children. Deficiencies or defects in the function of GH at, for example, receptor or signaling levels may result in short stature [1]. Insulin-like growth factor 1 (IGF-1) is a protein that mediates the action of GH. IGF-1 deficiency can disrupt the effects of GH and drastically impede muscle, bone, and overall growth [2].

There are two main forms of IGF-1 deficiency: primary and secondary. Primary IGF-1 deficiency is characterized by inadequate production of IGF-1 despite sufficient GH [3], while secondary IGF-1 deficiency includes other causes of low IGF-1, such as hypothyroidism, malnutrition, or growth hormone deficiency [2]. Severe primary IGF-1 deficiency (SPIGFD) is characterized by low basal IGF-1 levels, short stature, growth hormone sufficiency, and the exclusion of secondary forms of IGF-1 deficiency. SPIGFD results primarily from GH receptor mutations, post-receptor signaling defects, or inactive IGF-1 [3]. Clinical features of IGF-1 deficiency include short stature, low bone mineral density, and obesity or reduced lean body mass [1,4].

Since the recognition of IGF-1 deficiency as a primary endocrine disorder in the early 1990s, genetic mutations that cause low or inactive IGF-1 have been identified, such as mutations in the GH receptor gene in those with Laron syndrome [4]. These discoveries led to the development of treatments that bypass the mutated GH receptors, such as recombinant human IGF-1 (rhIGF-1) [2,5]. To date, research related to IGF-1 and SPIGFD has focused on the effectiveness, safety, and long-term effects of rhIGF-1 treatments [1,5,6,7], while details regarding its diagnosis and the prevalence of this ultra-rare disease are sparse.

The diagnosis of SPIGFD remains challenging, as secondary causes of IGF-1 deficiency exist [8]. Given the sparsity of reported cases of SPIGFD worldwide, we suspect that the condition is underdiagnosed, likely due to low IGF-1 levels being attributed to secondary causes. Reports of the prevalence of SPIGFD vary [9,10], and to our knowledge, the prevalence in Canada has not been previously reported. While rhIGF-1 is safe and effective in improving both linear growth rate and final adult height in children with SPIGFD [5,7,11], an electronic medical record (EMR) algorithm that could identify missed cases of SGPIFD does not exist. As a result, it is difficult to identify children that may benefit from rhIGF-1, potentially preventing affected children from receiving adequate therapy. The aim of our study was to establish a diagnostic algorithm using an electronic medical record system (EMR) and to determine the prevalence of SPIGFD among children with short stature at a large Canadian pediatric tertiary care center.

## 2. Materials and Methods

### 2.1. Study Design

We leveraged our EMR, Epic (Epic Systems, Verona, WI, USA), to identify our cohort and automate as much of the chart reviews as possible. To accomplish this, we developed an algorithm to identify patients with potential SPIGFD at the Children’s Hospital of Eastern Ontario (CHEO), Ottawa, ON, Canada. We obtained approval from the Research Ethics Board at CHEO. The requirement for informed consent was waived given the retrospective nature of the study and the fact that all the data collected were de-identified.

### 2.2. Study Population

We assessed the medical records of all children between the ages of 0 and 18 years with an outpatient encounter at CHEO between 1 November 2013 and 31 August 2021. Our inclusion criteria were based on diagnostic criteria for SPIGFD: short stature, defined as a height of <3.0 standard deviations (SD) below the mean for age and sex using the World Health Organization (WHO) growth charts for Canada [12], and low IGF-1, defined as basal levels < 2.5th percentile for sex, age, and/or pubertal status and/or bone age [13]. At our institution, we used two different IGF-1 assays: the IDS-iSYS assay (Immunodiagnostic Systems; Tyne & Wear, United Kingdom) from November 2013 to September 2017 and the Liaison assay (DiaSorin; Vercelli, Italy) as of October 2017. We defined IGF-1 values according to the appropriate assay and adjusted for pubertal stage and/or bone age as needed (depending on which one was available in the medical chart in cases where there was more than one low IGF-1 result based on pubertal stage or bone age, we used the first recorded instance of low). We defined pubertal status according to Tanner staging [14] and estimated bone age using the Greulich and Pyle atlas as reported by the reading pediatric radiologist [15]. 

### 2.3. Identification of Children with SPIGFD

The Epic algorithm we developed aimed to identify children meeting criteria for SPIGFD and exclude those with secondary IGF-1 deficiency, resulting in a limited number of manual chart reviews necessary to identify children with SPIGFD.

**Figure 1:**
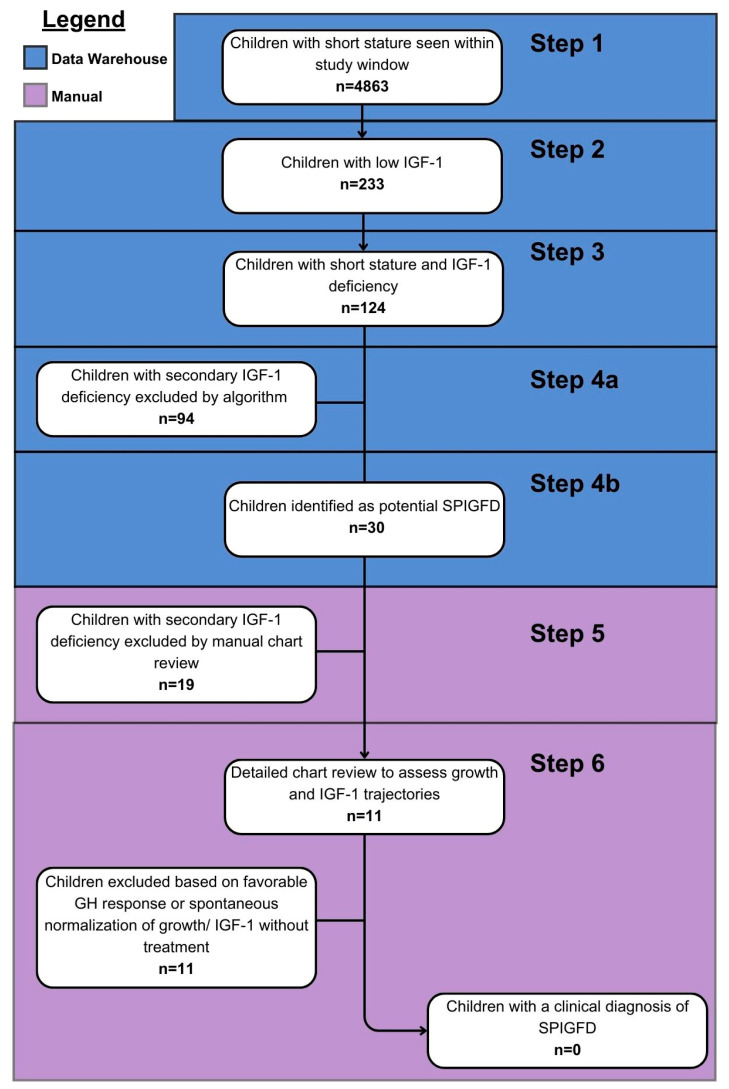
Flow chart of the algorithm to identify cases of SGPIFD via the Epic database, starting with all children seen at CHEO in our study time frame and ending with those meeting the diagnosis criteria for SPIGFD.

Our algorithm included six sequential steps (Figure 1). First, we identified all children with short stature (height ≤ −3.0 SD) based on the WHO growth charts for Canada [12] (step 1). Of those, we found the number of children with at least one IGF-1 value < 2.5th percentile for sex, age, and/or pubertal status and/or bone age (step 2). In cases where there was more than one low IGF-1 result, we used the first recorded instance of low IGF-1. In step 3, we identified the number of children with short stature recorded within one year of a low IGF-1 value, excluding those who either did not have an IGF-1 < 2.5th percentile or did not have a height recorded within 1 year of a low IGF-1. In step 4, we excluded children with secondary causes of IGF-1 deficiency, such as malnutrition, hypopituitarism, hypothyroidism, chronic treatment with supraphysiologic doses of steroids, liver disease, or other potential secondary causes for low IGF-1 values. We defined secondary causes of IGF-1 deficiency using the International Classification of Diseases 10th Revision (ICD-10) codes [16] for specific diagnoses (malnutrition, hypothyroidism, GH deficiency, and diseases of the liver) and body mass index (BMI) of <5th percentile on the WHO growth charts for Canada [12], suggestive of malnutrition. These diagnoses were taken from either the visit diagnoses or the problem lists; both EMR elements are frequently updated, and clinicians are unable to close encounters without indicating a visit diagnosis. A complete list of the ICD-10 codes used to define secondary causes of IGF-1 deficiency is available in Appendix A. We categorized the remaining children identified into six categories of abnormal growth, based on those defined by Teissier et al.: (1) transient pubertal slowing of growth with resumption of normal growth velocity at pubertal onset; (2) endocrine diseases (i.e., constitutional delay of growth, septo-optic dysplasia, etc.); (3) small for gestational age (as defined by length and/or weight at birth ≤ −2 SD); (4) genetic disorders (i.e., abnormal gene test results or multiple birth defects); (5) idiopathic short stature (ISS, as defined by height ≤ −2 SD with no identified etiology); and (6) adverse effects of medications on growth [9].

Clinical characteristics of patients identified with potential SPIGFD were verified via manual chart review to ensure we were only including children who met our criteria (i.e., we manually excluded children with GH deficiency, and secondary forms of IGF-1 deficiency not excluded by the algorithm; (step 5)). Growth hormone deficiency was defined as a suboptimal peak GH response to stimulation by clonidine and/or arginine. At our institution, we used two different analyzers with different peak GH definitions: peak value < 5.4 ng/mL using Dxl analyzers (Beckman Coulter; Brea, CA, USA) from 2005 to 27 November 2019, and peak value < 7.4 ng/mL using Roche analyzers (Hoffmann-La Roche, Basel, Switzerland) from 27 November 2019 onwards. Finally, we conducted an in-depth chart review, with 2 clinical experts (CB and MER) assessing the remaining patients for growth and IGF-1 trajectories to exclude any child who had a good height response to GH treatment (defined as an absolute height gain >0.5 SD within a 12-month period [17]), normalization of IGF-1 levels on GH treatment, or spontaneous normalization of IGF-1 and/or growth (step 6). 

### 2.4. Statistical Analysis

We used descriptive statistics to describe the proportion of children with potential SPIGFD identified by our Epic algorithm among children with short stature (height ≤ −3.0 SD) and low IGF-1 assessed at CHEO within the study period. All other clinical characteristics were similarly described using descriptive statistics. 

## 3. Results

### 3.1. Study Cohort

Figure 1 shows the results of our automated Epic algorithm and chart review. All patient encounters during our study window were screened, which yielded a total of 4863 unique children meeting our definition of short stature (step 1). Of the cohort, 233 (4.8%) had low IGF-1 (step 2), and 124 (2.5%) had both short stature and low IGF-1 within one year of each other (step 3). 

### 3.2. Description of Patients with Potential SPIGFD

**Figure 2:**
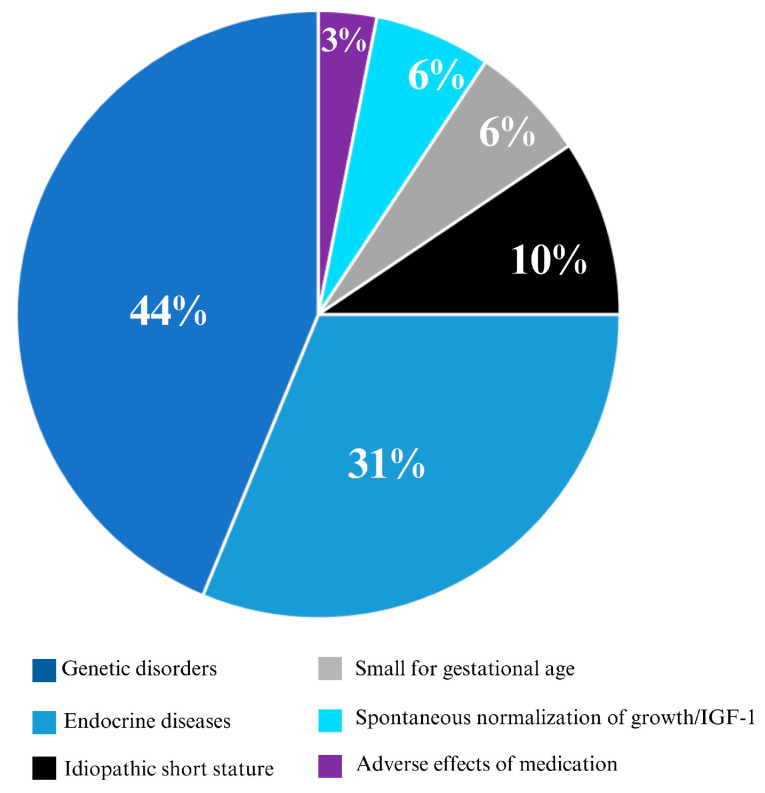
Categorization of children (*n* = 30) with potential SPIGFD identified by the Epic algorithm. One child had adverse effects of medications (chronic glucocorticoid use), 1 child had transient prepubertal slowing of growth, 2 children were small for gestational age, 3 children had idiopathic short stature, 8 children had endocrine diseases (6 with constitutional delay of growth and 2 with septic optic dysplasia), and 13 children had genetic disorders; 1 child had both an endocrine disease (constitutional delay of growth) and a genetic disorder (Duchenne Muscular Dystrophy), and 1 child had both an endocrine disease (constitutional delay of growth) and spontaneous normalization of growth/IGF-1.

Our Epic algorithm identified 30 children (0.6% of all children with short stature; 24% of all children with short stature and IGF-1 deficiency) with potential SPIGFD (step 4; Appendix A). The identified children (47% female) had a mean (SD) age of 7.0 (5.0) years, and mean (SD) height and weight z-scores of −3.5 (0.5) and −2.0 (1.5) at the time of their first low IGF-1 result. GH stimulation tests were normal in 47% of the cohort, and were not performed in the remaining 53%. The mean (SD) follow-up duration for these 30 children was 5.3 (1.7) years, and the cohort had a mean (SD) change in height SDS of 0.6 (2.0) over this time. The classification of these children is displayed in Figure 2. 

Of the 30 children identified by the algorithm with potential SPIGFD, we excluded 19 who were found to have secondary IGF-1 deficiency via the manual chart review (step 5). We assessed growth and IGF-1 trajectories in the remaining 11 children with potential SPIGFD, and all 11 patients were excluded: 5 children because of appropriate GH response, 4 due to spontaneous normalization of IGF-1, and 2 based on spontaneous normalization of both growth and IGF-1 (step 6) (Table 1). The median time to spontaneous IGF-1 normalization was 10.8 months (range 6.1–24.1 months). Ultimately, upon manual review, there were no children who met the criteria for a clinical diagnosis of SPIGFD during our study window.

## 4. Discussion

In this study, by leveraging our EMR, we created an electronic algorithm for characterizing the prevalence of SPIGFD. Through this algorithm, combined with minimal manual chart reviews, we determined that there were no children meeting criteria for a clinical diagnosis of SPIGFD among children with short stature at our large Canadian pediatric tertiary care center. While the algorithm identified 30 children with potential SPIGFD, representing a prevalence of 0.6% among children with short stature, we ultimately excluded all 30 patients upon manual chart review, resulting in a prevalence of 0%.

Out of 4863 children with short stature assessed, none met the diagnostic criteria for SPIGFD. Previous studies in France, England, and the United States have reported SPIGFD prevalence rates ranging from 1.2 to 25% using varied definitions and source populations [9,10,18,19]. For example, using a similar definition to that used in the present study (height < −3 SDS, serum IGF-1 levels < 2.5th percentile, GH sufficiency, and absence of causes of secondary IGF-1 deficiency), Teissier and colleagues found the prevalence of SPIGFD to be 0.2% in a large prospective cohort of French children with short stature [9]. Conversely, Edouard and colleagues found a SPIGFD prevalence of 20% in a small sample of prepubertal children with short stature, using a definition of height SDS ≤ −2.5, IGF-1 SDS ≤ −2, and GH sufficiency [10]. 

There may be several reasons that account for our finding of no children with SPIGFD relative to previous studies. First, we followed our cohort for an average of over 5 years, whereas previous studies followed cohorts for 6 months [9] or for an unspecified duration [10,18,19]. The importance of a longer duration of follow-up/documentation of the persistence of low IGF-1 is highlighted by the fact that the minimum time to spontaneous normalization of IGF-1 seen in our cohort was >6 months (median 10.8 months, range 6.1–24.1 months). While a single low IGF-1 value is insufficient to diagnose SPIGFD, it is expected that IGF-1 values should never normalize without rhIGF-1 therapy in the context of true SPIGFD. There is no consensus on the number of repeat assessments that should be conducted or over what time frame. There are also no internationally recognized standards regarding IGF-1 assay methodologies or sample collection and storage [20]. However, IGF-1 generation tests may be helpful in the diagnosis of SPIGFD [21].

It is unlikely that we may have missed cases of SPIGFD, as our institution is the only referral center for pediatric patients within our catchment area, and assessment of IGF-1 levels is part of our routine pediatric endocrinology workup for children with short stature. It is possible that some children meeting criteria for SPIGFD may have been seen in a clinic where IGF-1 testing is not necessarily routine, such as the genetics clinic. However, it is standard practice to refer children with short stature to endocrinology, where an IGF-1 is routinely performed. While some practice guidelines propose algorithms for when to test IGF-1 and/or treat with rhIGF-1 (e.g., [22], there is significant practice variation in this realm. Implementation of clear clinical algorithms guiding diagnosis and management would help to ensure all eligible children are referred and tested appropriately in the context of short stature. 

Third, unlike previous studies, which only assessed prepubertal children [9,10], we included all children regardless of pubertal status. It is possible that some children who initially presented as having potential SPIGFD may have normalized their IGF-1 during puberty, as was the case in our study (normalization of IGF-1 during puberty is not possible in cases of SPIGFD).

Our study has demonstrated that data stored in an EMR can successfully be leveraged to identify patients with rare diseases that are not easily classified using ICD codes. Data from EMRs have been successfully used to identify children with other complex conditions such as post-traumatic stress disorder [23], attention deficit hyperactivity disorders with psychiatric comorbidities [24], and adults with rare conditions such as allergic bronchopulmonary aspergillosis [25]. Automated algorithms and EMR data mining have been identified as potential ways to identify patients with rare diseases [26]. Leveraging clinical data to identify such patients has several advantages, including a reduction in the number of manual chart reviews required and the potential transferability to other centers using EMR. Another benefit is the potential to identify patients for relevant therapies or interventions, as would be the case with SPIGFD and the available treatment with rhIGF1. To our knowledge, this is the first time that an automated algorithm has been developed and used to identify children with SPIGFD. Leveraging our EMR resulted in fewer manual chart abstractions, which minimized reviewer time, and ensured standardization in the application of the diagnostic criteria throughout the algorithm steps, reducing potential bias. This proof-of-concept design will enable our methodology to be expanded to other pediatric tertiary care centers using a similar EMR. It will also allow for the potential for data from many centers to be easily pooled (with relatively minimal resource requirements), ultimately resulting in a larger population from which a definitive prevalence of SPIGFD could be determined. A large, multi-center sample may also provide an opportunity to utilize machine learning to develop a real-time clinical practice algorithm to identify which patients are likely to have SPIGFD and thus benefit from treatment with rhIGF-1. 

Our study has several strengths. To our knowledge, this is the largest cohort of children and the longest duration of follow-up included in a SPIFD prevalence study. Also, the problem lists and diagnoses in our EMR are frequently updated by clinicians and are therefore highly accurate, as clinicians are unable to close a clinical encounter or visit encounter without specifying a visit diagnosis. The results of our study should also be interpreted, given some potential limitations. Given the retrospective nature of the study, we were limited by the data available in health records, which may have resulted in some cases of SPIGFD being missed (for example, if an IGF-1 level was not completed in a patient with short stature). However, we cast a wide net with the intention of maximizing the sensitivity of our algorithm (by interpreting IGF-1 levels based on puberty and bone age rather than age only) in order to minimize the premature or incorrect exclusion of patients. Due to the ultrarare prevalence of SPIGFD, in order to maximize our chances of identifying a patient meeting these criteria, we utilized as wide of a date range as possible (i.e., from the earliest date EMR data were available until the time the work was completed). This left no room for a validation cohort, and therefore the algorithm could not be appropriately evaluated, which should be the subject of future work.

## 5. Conclusions

From a cohort of 4863 children with short stature, we identified no children with clinical SPIGFD. Though divergent from other prevalence studies, our findings are robust given our large sample size, long follow-up duration, and comprehensive chart reviews. The design, methodology, and results of this study will help inform future studies to identify potential cases of SPIGFD and ultimately help with the identification of children who may benefit from targeted therapy. A future study using a large, multi-center sample may also leverage our findings to utilize machine learning to develop a real-time clinical practice algorithm to identify which patients are likely to have SPIGFD and thus benefit from treatment with rhIGF-1. This model can also be replicated for other rare diseases.

## Figures and Tables

**Table 1:** Patients identified with potential SPIGFD after manual chart review for exclusion of secondary IGF-1 deficiency. Patients were assessed for GH response and spontaneous normalization of growth and IGF-1 levels (*n* = 11) (Step 5).

Study ID	Clinical Diagnosis	GH Treatment (Y/N)	GH Laboratory * and Clinical Treatment Response	Spontaneous Normalization of Growth (Y/N)	Spontaneous Normalization of IGF-1 (Y/N)
IGF22	ISS, constitutional delay, familial short stature	Y	Good laboratory and clinical response	N	N
IGF40	Genetic condition: 40 megabase deletion on chromosome 13	N	N/A	N	Y
IGF43	ISS, intrauterine growth restriction	Y	Good laboratory and clinical response	N	N
IGF50	ISS	N	N/A	N	Y
IGF05	ISS, constitutional delay	N	N/A	N	Y
IGF08	Familial short stature, constitutional delay	Y	Good laboratory response; poor clinical response	N	N
IGF49	Constitutional delay	N	N/A	N	Y
IGF37	Constitutional delay, transient prepubertal slowing of growth with resumption of normal growth velocity at puberty onset	N	N/A	Y	Y
IGF19	ISS	Y	Good laboratory and clinical response	N	N
IGF20	ISS, constitutional delay, familial short stature	Y	Good laboratory and clinical response	N	N
IGF39	Hemihypertrophy syndrome	N	N/A	Y	Y

Abbreviations: ISS—idiopathic short stature, GH—growth hormone; * Good laboratory response to GH defined as normalizing IGF-1 levels on GH therapy.

## Data Availability

The datasets presented in this article are not readily available because of ethical and privacy considerations. Requests to access the datasets or algorithm codes should be directed to the corresponding author.

## Appendix A

**Table A1:** Descriptive characteristics of children identified with potential SPIGFD by the Epic algorithm (*n* = 30) (Step 4).

Study ID	Diagnosis	Sex (M/F)	Age (years)	Height (SD)	Weight (SD)	GH Stimulation Test ^a^	Follow-Up Duration (years)	Change in Height (SDS)
IGF01	idiopathic short stature	F	15.5	−4.8	−3.3	Not performed	2.6	0.7
IGF02	Turner syndrome (non mosaic Turner syndrome variant)	F	15.0	−3.5	−1.7	Not performed	5.6	0.2
^b^ IGF04 ♦	constitutional delay	F	12.4	−3.1	−2.7	Normal	7.5	1.4
IGF05 ♦	idiopathic short stature + component of constitutional delay	M	13.7	−3.0	−1.0	Normal	4.1	0.4
^c^ IGF08 ♢	familial short stature + component of constitutional delay	M	13.2	−4.0	−2.5	Normal	4.1	1.1
IGF14	DMD on chronic glucocorticoids	M	8.5	−3.2	−0.7	Not performed	6.2	−1.3
IGF15 ♦	genetic condition: 22q11 deletion syndrome	M	8.3	−3.0	−2.4	Normal	6.3	1.1
IGF17	cleidocranial dysostosis	M	6.0	−3.4	−1.3	Not performed	7.1	1.4
IGF19 ♦	idiopathic short stature	F	5.8	−3.5	−3.0	Not performed	6.9	0.6
IGF20 ♦	idiopathic short stature + component of constitutional delay + component of familial short stature	F	5.4	−3.6	−2.4	Normal	7.2	0.9
IGF22 ♦	idiopathic short stature + component of constitutional delay + component of familial short stature, responded to GH	F	5.6	−3.1	−1.7	Normal	6.5	1.1
IGF24 ♦	idiopathic short stature born SGA	M	6.7	−3.2	−2.7	Normal	4.5	2.5
IGF26	septic optic dysplasia	F	4.8	−3.6	−2.1	Not performed	6.1	0.9
IGF27 ♦	genetic condition: caspase 8 deficiency	F	12.4	−3.1	−1.9	Normal	6.1	3.0
IGF28	genetic condition: phelan-mcdermid syndrome	F	2.4	−3.5	+0.2	Not performed	4.8	−0.5
IGF32	height spontaneously corrected	M	2.0	−3.4	−0.8	Not performed	4.4	2.7
IGF33	familial short stature & growth improved after QVAR d/c	M	3.1	−3.4	−2.2	Not performed	6.4	1.8
IGF35	McCune albright, no short stature currently–prev. did have a measurement −3std	M	3.7	−3.7	−4.0	Not performed	5.3	3.1
IGF36	septic optic dysplasia	F	3.9	−3.1	−2.7	Not performed	4.8	0.3
IGF37 ♦	constitutional delay-hx consistent, spontaneous improvement in growth velocity	M	12.0	−3.0	−1.7	Normal	4.4	0.8
IGF39	genetic: hemihypertrophy, normal growth velocity caught up to 3rd percentile; one measurement error prior to age 2 −3 std dev	F	2.2	−3.6	−1.0	Not performed	6.3	1.8
IGF40	genetic: 40 megabase deletion chromosome 13	F	0.7	−4.1	−3.1	Not performed	7.4	1.4
IGF41	Turner Syndrome	F	3.5	−3.1	−3.0	Not performed	7.1	−0.6
IGF43 ♦	idiopathic short stature/IUGR	M	6.1	−4.1	−2.3	Normal	0.5	1.2
IGF44	chronic glucocorticoids for autoimmune hemolytic anemia, disproportionate short stature	M	2.0	−3.4	−1.1	Not performed	5.6	−0.4
IGF45 ♦	genetic: SHOX deletion	M	16.4	−4.7	−3.3	Normal	3.7	−0.3
IGF46 ♦	spina bifida	M	3.0	−3.9	+3.6	Normal	5.7	−8.2
IGF49 ♦	constitutional delay	M	13.5	−3.2	−2.6	Normal	2.3	−0.1
IGF50 ♦	idiopathic short stature	F	0.7	−3.4	−3.7	Normal	5.4	−0.4
IGF51	nephrogenic DI-x linked, height improved spontaneously, 2017 ht and IGF-1 level meeting criteria	M	1.4	−3.8	−2.4	Not performed	4.0	2.5

All patients had IGF-1 levels < 2.5th percentile for age, sex, and/or pubertal stage or bone age. All parameters were measured at the first reported low IGF-1 value, except the ‘Follow-up duration’ and ‘Change in height’. ^a^ GH response was defined as a peak growth hormone response to stimulation by clonidine and/or arginine (normal response defined as a peak value greater than 5.4 ng/mL from date 2005 to 27 November 2019, using Beckman Dxl analyzers and greater than 7.4 ng/mL from 27 November 2019 onwards, using Roche analyzers). ^b^ Children who had a good response to growth hormone treatment: ♦. ^c^ Children who had a poor response to growth hormone treatment: ♢.

## Appendix B

Complete list of the ICD-10 codes used to define secondary causes of IGF-1 deficiency:

Malnutrition-related ICD-10 codes
E40→Kwashiorkor
E41→Nutritional marasmus
E42→Marasmic kwashiorkor
E43→Unspecified severe protein-energy malnutrition
E44→Protein-energy malnutrition of moderate and mild degree
E44.0→Moderate protein-energy malnutrition
E44.1→Mild protein-energy malnutrition
E45→Retarded development following protein-energy malnutrition
E46→Unspecified protein-energy malnutrition

Hypothyroidism-related ICD-10 codes
E03.0→Congenital hypothyroidism with diffuse goitre
E03.1→Congenital hypothyroidism without goitre
E03.2→Hypothyroidism due to medicaments and other exogenous substances
E03.3→Postinfectious hypothyroidism
E03.5→Myxoedema coma
E03.8→Other specified hypothyroidism
E03.9→Hypothyroidism, unspecified

Growth Hormone Deficiency-related ICD-10 codes
E23.0→Hypopituitarism

Disease of Liver-related ICD-10 codes
Q44.6→Cystic disease of liver
K70.0→Alcoholic fatty liver
K70.1→Alcoholic hepatitis
K70.2→Alcoholic fibrosis and sclerosis of liver
K70.3→Alcoholic cirrhosis of liver
K70.4→Alcoholic hepatic failure
K70.9→Alcoholic liver disease, unspecified
K71.0→Toxic liver disease with cholestasis
K71.1→Toxic liver disease with hepatic necrosis
K71.2→Toxic liver disease with acute hepatitis
K71.3→Toxic liver disease with chronic persistent hepatitis
K71.4→Toxic liver disease with chronic lobular hepatitis
K71.5→Toxic liver disease with chronic active hepatitis
K71.6→Toxic liver disease with hepatitis, not elsewhere classified
K71.7→Toxic liver disease with fibrosis and cirrhosis of liver
K71.8→Toxic liver disease with other disorders of liver
K71.9→Toxic liver disease, unspecified
K72.0→Acute and subacute hepatic failure
K72.1→Chronic hepatic failure
K72.9→Hepatic failure, unspecified
K73.0→Chronic persistent hepatitis, not elsewhere classified
K73.1→Chronic lobular hepatitis, not elsewhere classified
K73.2→Chronic active hepatitis, not elsewhere classified
K73.8→Other chronic hepatitis, not elsewhere classified
K73.9→Chronic hepatitis, unspecified
K74.0→Hepatic fibrosis
K74.1→Hepatic sclerosis
K74.2→Hepatic fibrosis with hepatic sclerosis
K74.3→Primary biliary cirrhosis
K74.4→Secondary biliary cirrhosis
K74.5→Biliary cirrhosis, unspecified
K74.6→Other and unspecified cirrhosis of liver
K75.0→Abscess of liver
K75.2→Nonspecific reactive hepatitis
K75.3→Granulomatous hepatitis, not elsewhere classified
K75.4→Autoimmune hepatitis
K75.8→Other specified inflammatory liver diseases
K75.9→Inflammatory liver disease, unspecified
K76.0→Fatty (change of) liver, not elsewhere classified
K76.1→Chronic passive congestion of liver
K76.2→Central haemorrhagic necrosis of liver
K76.3→Infarction of liver
K76.4→Peliosis hepatis
K76.7→Hepatorenal syndrome
K76.8→Other specified diseases of liver
K76.9→Liver disease, unspecified
K77.0→Liver disorders in infectious and parasitic diseases classified elsewhere
K77.8→Liver disorders in other diseases classified elsewhere

## References

[1] Backeljauw P.F., Kuntze J., Frane J., Calikoglu A.S., Chernausek S.D. (2013). Adult and Near-Adult Height in Patients with Severe Insulin-Like Growth Factor-I Deficiency after Long-Term Therapy with Recombinant Human Insulin-Like Growth Factor-I. Horm. Res. Paediatr..

[2] Backeljauw P.F., Chernausek S.D. (2006). Treatment of Insulin-Like Growth Factor Deficiency with IGF-I: Studies in Humans. Horm. Res..

[3] Moore B., Whitehead A., Davies K., Llahana S., Follin C., Yedinak C., Grossman A. (2019). Short stature, growth hormone deficiency, and primary IGF-1 deficiency. Advanced Practice in Endocrinology Nursing.

[4] Laron Z. (2004). Laron Syndrome (Primary Growth Hormone Resistance or Insensitivity): The Personal Experience 1958–2003. J. Clin. Endocrinol. Metab..

[5] Chernausek S.D., Backeljauw P.F., Frane J., Kuntze J., Underwood L.E. (2007). Long-Term Treatment with Recombinant Insulin-Like Growth Factor (IGF)-I in Children with Severe IGF-I Deficiency due to Growth Hormone Insensitivity. J. Clin. Endocrinol. Metab..

[6] Cohen J., Blethen S., Kuntze J., Smith S.L., Lomax K.G., Mathew P.M. (2014). Managing the Child with Severe Primary Insulin-Like Growth Factor-1 Deficiency (IGFD): IGFD Diagnosis and Management. Drugs R D.

[7] Bang P., Woelfle J., Perrot V., Sert C., Polak M. (2021). Effectiveness and Safety of rhIGF1 Therapy in Patients with or without Laron Syndrome. Eur. J. Endocrinol..

[8] Ranke M.B. (2006). Defining Insulin-Like Growth Factor-I Deficiency. Horm. Res..

[9] Teissier R., Flechtner I., Colmenares A., Lambot-Juhan K., Baujat G., Pauwels C., Samara-Boustani D., Beltrand J., Simon A., Thalassinos C. (2014). Characterization and Prevalence of Severe Primary IGF1 Deficiency in a Large Cohort of French Children with Short Stature. Eur. J. Endocrinol..

[10] Edouard T., Grünenwald S., Gennero I., Salles J.P., Tauber M. (2009). Prevalence of IGF1 Deficiency in Prepubertal Children with Isolated Short Stature. Eur. J. Endocrinol..

[11] Kemp S.F. (2009). Insulin-Like Growth Factor-I Deficiency in Children with Growth Hormone Insensitivity: Current and Future Treatment Options. BioDrugs.

[12] WHO WHO Growth Charts for Canada 2014. https://www.dietitians.ca/Secondary-Pages/Public/Who-Growth-Charts.aspx.

[13] Collett-Solberg P.F., Misra M., Society TCotLWPE (2008). The Role of Recombinant Human Insulin-Like Growth Factor-I in Treating Children with Short Stature. J. Clin. Endocrinol. Metab..

[14] Tanner J.M., Whitehouse R.H. (1976). Clinical Longitudinal Standards for Height, Weight, Height Velocity, Weight Velocity, and Stages of Puberty. Arch. Dis. Child..

[15] Greulich W.W., Pyle S.I. (1959). Radiographic Atlas of Skeletal Development of the Hand and Wrist.

[16] Brämer G.R. (1988). International Statistical Classification of Diseases and Related Health Problems. Tenth Revision. World Health Stat. Q..

[17] Ranke M.B., Lindberg A., Cowell C.T., Wikland K.A., Reiter E.O., Wilton P., Price D.A., KIGS International Board (2003). Prediction of Response to Growth Hormone Treatment in Short Children Born Small for Gestational Age: Analysis of Data from KIGS (Pharmacia International Growth Database). J. Clin. Endocrinol. Metab..

[18] Attie K.M., Julius J.R., Stoppani C., Rundle A.C. (1997). National Cooperative Growth Study Substudy VI: The Clinical Utility of Growth-Hormone-Binding Protein, Insulin-Like Growth Factor I, and Insulin-Like Growth Factor-Binding Protein 3 Measurements. J. Pediatr..

[19] Clayton P.E., Ayoola O., Whatmore A.J. (2006). Patient Selection for IGF-I Therapy. Horm. Res..

[20] Frystyk J., Freda P., Clemmons D.R. (2010). The Current Status of IGF-I Assays—A 2009 Update. Growth Horm. IGF Res..

[21] Cotterill A., Camacho-Hübner C., Woods K., Martinelli C., Duquesnoy P., Savage M. (1994). The Insulin-Like Growth Factor I Generation Test in the Investigation of Short Stature. Acta Paediatr..

[22] Grimberg A., DiVall S.A., Polychronakos C., Allen D.B., Cohen L.E., Quintos J.B., Rossi W.C., Feudtner C., Murad M.H., Drug and Therapeutics Committee and Ethics Committee of the Pediatric Endocrine Society (2016). Guidelines for Growth Hormone and Insulin-Like Growth Factor-I Treatment in Children and Adolescents: Growth Hormone Deficiency, Idiopathic Short Stature, and Primary Insulin-Like Growth Factor-I Deficiency. Horm. Res. Paediatr..

[23] Zafari H., Kosowan L., Zulkernine F., Signer A. (2021). Diagnosing Post-Traumatic Stress Disorder Using Electronic Medical Record Data. Health Inf. J..

[24] Slaby I., Hain H.S., Abrams D., Mentch F.D., Glessner J.T., Sleiman P.M.A., Hakonarson H. (2022). An Electronic Health Record (EHR) Phenotype Algorithm to Identify Patients with Attention Deficit Hyperactivity Disorders (ADHD) and Psychiatric Comorbidities. J. Neurodev. Disord..

[25] Maguire A., Johnson M.E., Denning D.W., Ferreira G.L., Cassidy A. (2017). Identifying Rare Diseases Using Electronic Medical Records: The Example of Allergic Bronchopulmonary Aspergillosis. Pharmacoepidemiol. Drug Saf..

[26] Garcelon N., Burgun A., Salomon R., Neuraz A. (2020). Electronic Health Records for the Diagnosis of Rare Diseases. Kidney Int..

